# The Potential of Black Radio to Disseminate Health Messages and Reduce Disparities

**Published:** 2010-06-15

**Authors:** Ingrid J. Hall, C. Ashani Johnson-Turbes, Kymber N. Williams

**Affiliations:** Centers for Disease Control and Prevention; ICF Macro, Atlanta, Georgia; Centers for Disease Control and Prevention, Atlanta, Georgia

## Abstract

Radio stations that target African American audiences ("black radio") reach a national African American audience daily, making black radio an ideal medium for health promotion and disparities reduction in the African American community. Black radio can be used to communicate public health messages and to recruit African Americans into public health research.

## Introduction

Most urban areas in the United States have radio stations that target and reach African American audiences ("black radio"). Such stations typically devote a substantial percentage of air time to programming such as call-in shows, personal on-air interviews, and community promotions, rather than news and public affairs programming (1). Black radio is an important communication channel to reach African American audiences and can play a role in health promotion in the African American community (1-5). Black radio stations can be effective change agents by encouraging community partnerships and promoting drug awareness, nonviolent behavior, education, and other community issues, including those related to health (1).

## Radio Use by African Americans

Black radio reaches a broad cross-section of the black community (youths, the elderly, women, men, young adults, and all income groups). The "black community" in the context of radio encompasses a globally diverse group, including African Americans, Haitians, Africans, and Caribbeans. Media marketing reports document the consistency of radio use among African Americans of all ages (6). For example, more than 90% of African American consumers aged 12 years or older listen to the radio weekly, a higher penetration rate than that of television, magazines, newspapers, or the Internet (6).

Marketing studies also show that African Americans listen to the radio in diverse environments, including at home, at work, in the car, in stores and restaurants, online, and more recently via mobile telephones (cell phones) (6). One such study found that African American radio listeners spend, on average, more than 3 hours per day listening to the radio (6). In most demographic segments, listeners spend more than 21 hours per week listening to radio. African American men and women aged 45 to 64 years spend the most time listening to the radio, tuning in more than 24 hours per week. More than 94% of African Americans older than 55 listen to the radio at least once per week (6).

Urban African American radio listeners use the radio as a source of information. A national survey of 1,895 media users of various languages and racial/ethnic backgrounds found that 58% of African American respondents use ethnic radio to obtain information (7). Among African American respondents, 37% indicated that it is a favored information source and 21% reported using ethnic radio for information in addition to mainstream sources (7).

## Using Black Radio for Recruitment Into Public Health Research

One barrier to the participation of African Americans in health promotion and intervention research and services is suspicion and mistrust of public health researchers because of a documented history of mistreatment of African Americans (3,8,9). Black radio's status as a long-trusted source of information makes it an effective way to recruit African Americans into intervention studies. A 2009 study of recruitment methods found that reactive methods (those requiring the participant to contact study personnel) using television, radio, or newspaper ads were more effective for African American recruitment (43% of enrolled) than proactive (face-to-face) methods. Radio was the most effective reactive tool, accounting for 20% of the total study sample (10).

One study successfully used paid radio advertisements to recruit low-income, African American women into focus groups to explore attitudes and beliefs related to breast cancer and mammography (11-13). In another study, radio was effectively used to recruit African American preteens into a multisite obesity prevention trial. This study found that across sites, 29% of caregivers of the children were more likely to hear about the program from the radio than from other sources (2). In some sites, as many as 65% of caregivers of program enrollees heard about the study on the radio. A study of recruitment methods to enroll men into a longitudinal prostate cancer screening study found that more men were recruited via radio than by referral or other methods (14). Most of these men were African American, poorly educated, and of low socioeconomic status. Taken together, these studies show the appeal of using of this communication medium to reach African Americans.

Black radio has also proved effective in promoting specific public health programs. Radio was the most effective venue for reaching African American men with information about a community-based screening program, whereas newspapers were most effective for reaching white men (3).  In a study of recruitment strategies used to enroll participants into a multicenter trial of lifestyle interventions for blood pressure control, mass distribution of brochures was more effective with non-Hispanic whites, but African Americans responded better to other recruitment strategies, including radio (4). Additionally, radio was a component of a mass media campaign in New Orleans designed to promote walking and fruit and vegetable consumption in a low-income, predominantly African American urban population (5). After 5 months, substantial increases were seen over baseline in measures of message recall, positive attitudes toward fruit and vegetable consumption, and positive attitudes toward walking (5).

## Using Contemporary Black Radio to Communicate Public Health Messages

Since the mid-1990s, black radio has transitioned from individual, locally owned stations to large networks of stations owned by a handful of major companies. These networks have allowed influential, nationally syndicated radio personalities, such as Tom Joyner, to be heard across the country daily. The "Tom Joyner Morning Show" reaches more than 8 million listeners in more than 115 markets each week (15). Two newcomers to syndication, Steve Harvey and Michael Baisden, also reach many listeners: Steve Harvey's show airs in 64 markets (5.9 million listeners weekly), and Michael Baisden's show airs in 58 markets (4.6 million listeners weekly) (Alexandra Fenech, Premier Radio, e-mail communication, March 11, 2009). These personalities also have an online presence that includes social networks for their listening audiences. Joyner's BlackAmericaWeb.com has a growing Web presence (approximately 750,000 members and 30,000 unique visitors per month) (15), as does Baisden's MingleCity.com (40 million visits per month) (16). These modern-day black radio pioneers, among others, can raise the political, social, and health consciousness of African Americans nationally.

Syndicated black radio has been used much less frequently to disseminate health messages than to inform listeners of political and social issues. However, 2 notable examples illustrate its potential. First, "Take a Loved One to the Doctor Day" is an annual event that urges people to take themselves or family members to a doctor for a health screening (17). The campaign is designed to reduce health disparities and to improve access to culturally relevant health care information. The campaign also seeks to link African Americans to the federal government's extensive health information resources. Throughout the month before the event, ABC Radio Networks broadcasts educational segments on chronic diseases that disproportionately affect African Americans (eg, cancer, diabetes, heart disease).

The second major public health initiative using syndicated urban radio for marketing and outreach is the "50 Million Pound Challenge" (www.50millionpoundchallenge.com). Created by Ian K. Smith, MD, in early 2007, this initiative encourages African Americans to overcome obesity by highlighting the high prevalence of overweight among African American men (67%), women (80%), and adolescents (20%) and the health challenges associated with overweight and obesity (18). To promote healthy nutrition and physical activity, Dr Smith regularly appears on the Joyner, Harvey, and Baisden shows as part of a multicomponent, multimedia promotional campaign. The campaign recently teamed up with *The Steve Harvey Morning Show* in a joint online venture. Listeners can follow the same diet plan as Steve Harvey. From April 2007 through March 2009, more than 950,000 people enrolled in the Challenge online. Program organizers estimate that 35% to 45% of enrollees became aware of the Challenge through black radio (Ian Smith, MD, e-mail communication, March 12, 2009).

## Black Radio and Public Health

The prevalence of cancer, heart disease, stroke, and diabetes is disproportionately high among African Americans. Reaching this population with public health messages that address African Americans' lack of knowledge and awareness of preventive health behaviors will require comprehensive communication strategies (19,20). Black radio, a culturally tailored community information resource, could prove effective in filling this need.

Low health literacy — the inability to read, understand, and use health care materials — is consistently associated with race/ethnicity, and studies show that African Americans have lower health literacy than whites. Low health literacy is associated with worse health outcomes and poorer health status (21,22). For example, low health literacy among African American men is associated with diagnosis of prostate cancer (23). Authors concluded that low literacy may be a barrier to the diagnosis of early-stage prostate cancer among this population and recommended the development of culturally appropriate, low-literacy materials to improve diagnosis of early-stage prostate cancer. Similarly, another study recommended the development of culturally appropriate, low-literacy smoking cessation materials for African American and Hispanic women to successfully promote tobacco abstinence (24). Black radio has advantages over print media for circumventing low health literacy.

Low health literacy has been associated with less knowledge of preventive measures, misunderstanding about risk, failure to appreciate the benefits of early detection, and lack of knowledge about available treatments for cancer (25). Low health literacy may influence choice of information source, how well information is absorbed, and how complex messages can be while still being effectively conveyed. Given the prevalence in the United States of low literacy in general and low health literacy in particular among elderly and minority populations (25), print media may not be the most effective way to influence these groups. An intervention that added an easy-to-read printed brochure to a physician recommendation for a mammogram was no more effective at increasing screening mammography rates than the recommendation alone among a low-literacy audience (26). Older African American men were asked how they preferred to receive messages about prostate cancer screening (27). Although respondents sometimes considered printed information such as newsletters and brochures to be helpful, on the whole, they did not view print materials as the best way to communicate health information. Participants said that talking with others about prostate cancer and finding out about screening practices through "word-of-mouth" were the most effective strategies for reaching African American men with prostate cancer prevention messages (27). Black radio supports the strategy of "word-of-mouth" dissemination by trusted sources.

Call-in shows and personal on-air interviews are formats unique to radio that can be effective for promoting social learning, whereby people reciprocally learn from each other. Messages on such shows can be tailored to target audiences and designed to contain interactive elements customized to local community concerns. Radio provides a forum for 2-way communication via live radio shows, during which listeners can engage messengers and each other to obtain health and other information. In a recent study on mammography promotion, social learning and listener interaction via radio was an effective way to disseminate health information (11-13). In this study, on-air discussions between a moderator, health professional, and breast cancer survivor prompted several calls to the local radio station from listeners and increased calls during the next several months to a national helpline to inquire about low-cost mammograms for low-income women (Ingrid J. Hall, African American Women and Mass Media Study, Centers for Disease Control and Prevention, 2009, unpublished data).

Although some researchers have identified television as a major source of health information, studies have shown that television messages raise awareness but do not educate or adequately inform audiences (25). In contrast, radio can provide an interactive forum that focuses on education, patient action, motivation, and self-empowerment (28-30). In one study, African American community members and radio listeners trusted black radio to "talk their talk" and were enthusiastic about interactive participation in black radio's conversational activities (30). This study supports the notion that black radio (especially talk or conversational radio) is an empowerment tool for public discourse in the African American community.

Shows such as "The Michael Baisden Show" provide this sort of interactive and conversational platform for discussing social, political, and economic issues. Black radio also serves as a forum for valuable "everyday talk" that remains an uncultivated dimension of communication for eliminating health disparities (19). In this way, black radio is not only a communication channel or strategy as defined by traditional health communication literature but also a trusted communication source for information about issues such as politics, economics, racial identity, community, and health. Because the roots of health disparities extend into socioeconomic and political conditions (30), black radio, as a forum for dissemination of health messages and deliberation about health concerns, holds promise as a communication channel and information source to eliminate health disparities.

## Implications for Practice

The viability of community communication channels able to target loyal audiences makes black radio a good choice for public health practitioners. Black radio is a communication channel with an established audience of African American listeners, making it a valuable resource for reaching African Americans with health messages, even in an environment characterized by increased attention to new media (eg, mobile communication devices — PDAs [personal digital assistants], cell phones). Public health practitioners should consider using a wider range of media and approaches to change behavior and further explore how black radio — a potentially cost-effective communication channel, along with its online networks and supportive community partnerships — can be used and evaluated as a method of communicating to African American audiences.
